# Genome-Wide Evaluation of Histone Methylation Changes Associated with Leaf Senescence in Arabidopsis

**DOI:** 10.1371/journal.pone.0033151

**Published:** 2012-03-12

**Authors:** Judy A. Brusslan, Ana M. Rus Alvarez-Canterbury, Nishanth Ulhas Nair, Judd C. Rice, Michael J. Hitchler, Matteo Pellegrini

**Affiliations:** 1 Department of Biological Sciences, California State University Long Beach, Long Beach, California, United States of America; 2 Laboratory for Computational Biology and Bioinformatics, School of Computer and Communication Sciences, Ecole Polytechnique Fédérale de Lausanne, Lausanne, Switzerland; 3 Biochemistry and Molecular Biology, University of Southern California, Norris Cancer Center, Los Angeles, California, United States of America; 4 Radiation Oncology Department, Kaiser Permanente, Los Angeles, California, United States of America; 5 Department of Molecular, Cell and Developmental Biology, University of California Los Angeles, Los Angeles, California, United States of America; National Cancer Institute, United States of America

## Abstract

Leaf senescence is the orderly dismantling of older tissue that allows recycling of nutrients to developing portions of the plant and is accompanied by major changes in gene expression. Histone modifications correlate to levels of gene expression, and this study utilizes ChIP-seq to classify activating H3K4me3 and silencing H3K27me3 marks on a genome-wide scale for soil-grown mature and naturally senescent Arabidopsis leaves. ChIPnorm was used to normalize data sets and identify genomic regions with significant differences in the two histone methylation patterns, and the differences were correlated to changes in gene expression. Genes that showed an increase in the H3K4me3 mark in older leaves were senescence up-regulated, while genes that showed a decrease in the H3K4me3 mark in the older leaves were senescence down-regulated. For the H3K27me3 modification, genes that lost the H3K27me3 mark in older tissue were senescence up-regulated. Only a small number of genes gained the H3K27me3 mark, and these were senescence down-regulated. Approximately 50% of senescence up-regulated genes lacked the H3K4me3 mark in both mature and senescent leaf tissue. Two of these genes, *SAG12* and At1g73220, display strong senescence up-regulation without the activating H3K4me3 histone modification. This study provides an initial epigenetic framework for the developmental transition into senescence.

## Introduction

*Arabidopsis thaliana*, similar to most crop plants, is a monocarpic species that undergoes whole plant senescence to maximize energy input into reproductive structures. Understanding the molecular mechanisms of nutrient recycling during leaf senescence is critical for the production of high yielding crops. Global gene expression has been characterized in senescent Arabidopsis tissue [1], [2], [3], [4], and senescence up-regulated genes (SURGs) are enriched for autophagy, response to reactive oxygen species, chlorophyll and lipid catabolism as well as carbohydrate and nitrogen transport. Senescence down-regulated genes (SDRGs) are enriched for cytoplasmic protein synthesis as well as chloroplast functions such as light harvesting, carbon fixation, and photorespiration. A leaf senescence database compiles senescence associated genes and orthologs from numerous plant species [5]. Transcription factors that play a role in these global changes in gene expression have been identified. Genetic and biochemical evidence support a role for WRKY53 (Locus:2128514) and NAC family members VNI2 (Locus:2179877) and AtNAP (Locus:2007166) as positive regulators of senescence [6], [7], [8], [9], [10] , and microarray analysis reveals over-representation for the AP2-EREBP, bZIP, C3H, CCAAT, NAC and WRKY transcription factor families as senescence progresses [1].

Transcription factors can recruit histone modifying enzymes to specific promoter regions [11] and alterations in histone structure have been shown to be important for the vegetative to reproductive transition in Arabidopsis [12]. The floral repressor gene, *FLC* (Locus:2184118), is silenced during vernalization, a 40 day cold treatment, when activating H2BK143ub1, H3K4me3 and H3K36me3 histone marks are removed from *FLC* and replaced with silencing H3R3sme, H3K9me3 and H3K27me3 histone marks. *FLC* is reactivated in the globular stage of embryogenesis in a process that requires replacement of H2A with the H2AZ histone variant [13]. Endosperm development is regulated via genomic imprinting which utilizes histone modification and DNA methylation to silence maternal or paternal alleles [14]. In rice, expression of genes important for brassinosteroid synthesis and signaling are dependent on SDG725, an H3K36 methyltransferase [15]. In addition to development, histone modifications accompany changes in gene expression in response to deetiolation [16] and abiotic stresses including drought, cold, salt and hypoxia [17].

Dynamic chromatin modifications may also be important for the changes in gene expression associated with senescence. Decondensation of heterochromatin occurs in the early stages of Arabidopsis leaf senescence and overexpression of SUVH2 (Locus:2051083), an H3K9 methyltransferase which promotes the formation of ectopic heterochromatin, has pleiotropic phenotypic effects which include a delay in senescence [18]. Low-level overexpression of a chromatin-modifying AT-hook protein, ORE7/ESC (Locus:2037350), also resulted in delayed senescence [19]. In yeast, worms, flies and mammals, the sirtuin family of histone deacetylases function to prevent early senescence by deacetylation of H3K9 in a suite of promoters controlled by NF-κB [20]. Although the mechanism has not been reported, the Arabidopsis histone deacetylase mutant, *hda6* (Locus:2162017), also has increased leaf longevity [21].

This work focuses on two histone modifications, H3K4me3 and H3K27me3. Trimethylation of H3K4 is catalyzed by the COMPASS-like protein complexes which form a scaffold for different SET-domain methyltransferases [22]. H3K4me3 marks have been evaluated on a genome-wide scale using chromatin immunoprecipitation (ChIP) followed by hybridization to high-density genomic arrays [23], and found to associate at and just downstream of the transcription start site (TSS) of expressed genes. This is similar to humans where H3K4me3 is associated with active genes near the TSS [24]. In Arabidopsis, the Pol II-associating factor 1 complex (Paf1C) guides H3K4me3 marks to the 5′-end of a subset of actively transcribed genes by linking elongating Pol II with histone methyltransferases after ubiquitination of H2B [25], however this mechanism is not used for activation of all genes since H2BK143Ub1 precedes H3K4 trimethylation for some, but not all, Arabidopsis genes [26].

Trimethylation of H3K27 in plants, is catalyzed by the Polycomb-group protein complex 2 (PRC2) [27], [28], and is found to be associated with highly tissue-specific genes when they are silenced. H3K27me3 is enriched within the body of the gene from 25% to 75% of gene length [29]. For some embryonic and stem cell regulator genes, H3K27me3 is recognized by a plant version of PRC1 which subsequently monoubiquitinates H2AK120 and aids in gene silencing [30]. The H3K27me3 histone modification plays an important role in controlling the size of the floral meristem [31] and in leaf development [32].During vernalization, the *COLDAIR* noncoding intronic RNA, which is encoded by the first intron of *FLC*, targets PRC2 to the *FLC* locus to promote trimethylation of H3K27 and silencing of the floral repressor [33].

In this study, genome-wide locations of H3K4me3 and H3K27me3 marks in mature and senescent Arabidopsis leaves were determined by ChIP-seq. Genomic regions that showed significant differences in these modifications were identified using ChIPnorm and correlated to changes in gene expression that accompany senescence. Gain or loss of the activating H3K4me3 mark correlated to up- or down-regulation during senescence and similar, but opposite, trends were observed for the silencing H3K27me3 mark. This loss in H3K4 trimethylation in older tissue was accompanied by an increase in expression of *KDM5B-like* genes encoding H3K4 demethylases. Surprisingly, two genes that were strongly up-regulated during senescence completely lacked the activating H3K4me3 mark.

## Results

### H3K4me3 and H3K27me3 marks in mature and senescent leaves

**Figure 1:**
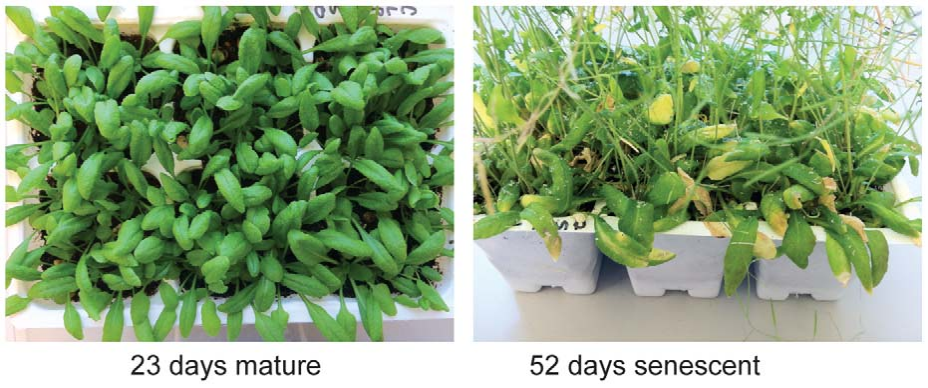
Mature (23 d) and senescent (52 d) leaf tissue used for ChIP-seq and RNA isolation. Fully-expanded, green rosette leaves were harvested from mature and senescent plants similar to those shown above.

Arabidopsis plants, grown on soil in light chambers with continuous illumination, were used in this study. Mature leaves were harvested 23 days after germination (23 d) when plants had incipient bolts and large expanded green rosette leaves. Senescent leaves were obtained from 52 day-old (52 d) plants with elongated bolts in which about half of the siliques were brown and dry (Figure 1). The rosettes of the older plants had yellow-green leaves near the soil, but only fully-expanded green leaves were harvested. No cauline leaves or newly developed rosette leaves were harvested.

**Figure 2:**
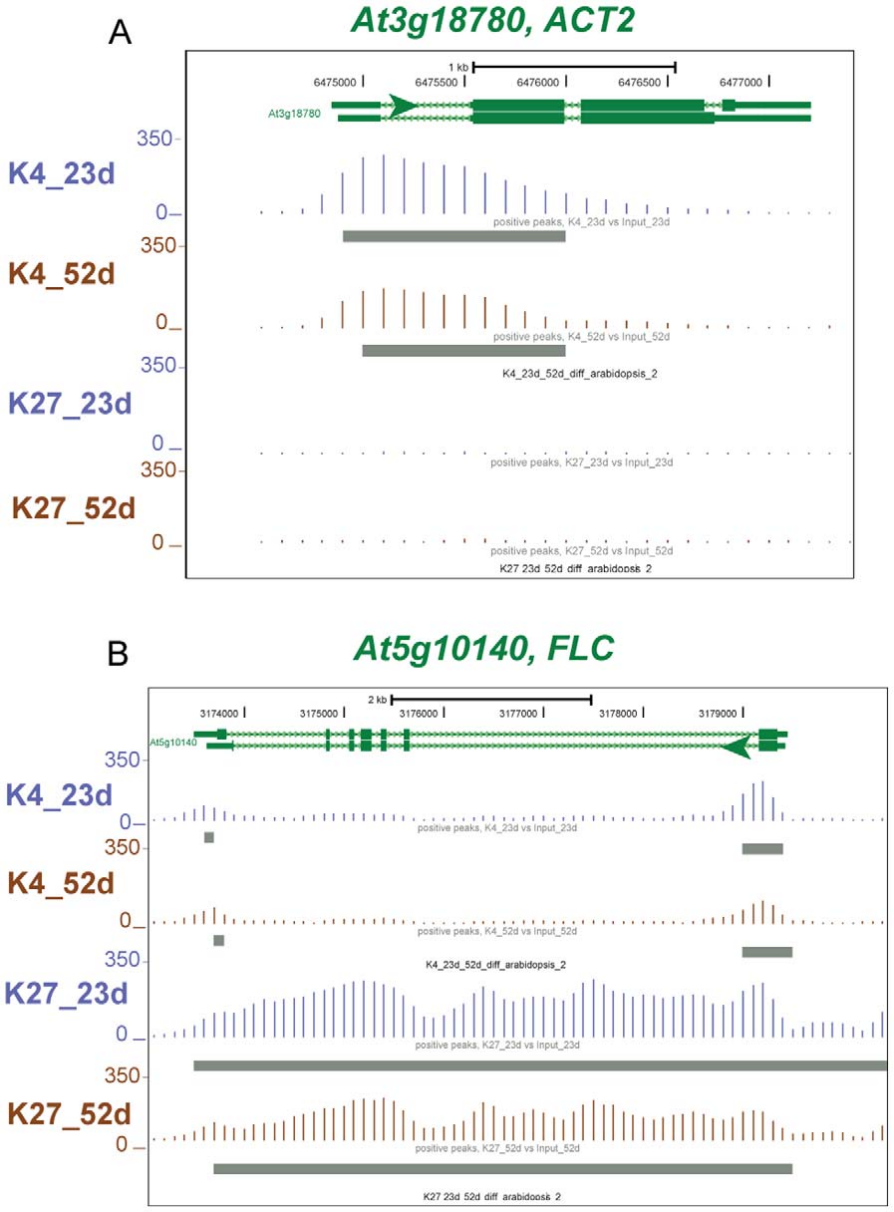
Expected patterns of H3K4me3 and H3K27me3 histone modifications on two representative genes. Genes with exons represented as green thick lines and introns shown as green thin lines are shown at the top of this Genome Browser image, and the green arrow shows the direction of transcription. The number of sequence reads for 23 d are shown in blue and for 52 d in brown. H3K4me3 (K4) is shown on the top two tracks, H3K27me3 (K27) on the next two tracks. Gray rectangles below tracks indicate regions with sequence reads significantly above relative input. A) *ACT2* (*At3g18780*) shows significant enrichment above respective input samples for the H3K4me3 mark. This constitutive gene is associated with H3K4me3 marks, but lacks H3K27me3 marks. B) *FLC* (*At5g10140*) shows significant levels of silencing H3K27me3 marks, which is expected in the *fri* mutant Columbia ecotype that does not require vernalization for flowering. *FLC* has some H3K4me3 marks near the first and last exons.

Chromatin immunoprecipitation was performed using antibodies that recognize H3K4me3, H3K27me3, and H3-general along with input samples. Approximately 20 million unique single end reads were obtained for H3-general and input samples and the H3K4me3_23d sample while approximately 13 million unique single end reads were obtained for the two H3K27me3 samples (_23d and _52d) and the H3K4me3_52d sample. For all subsequent analyses, the appropriate input sample was chosen as the control since input reads did not significantly differ from H3-gen reads (Figure S1). Data are displayed on a mirror UCSC genome browser (http://genomes.mcdb.ucla.edu/) under Senescence ChIP-seq and tracks are displayed in the following order, from top to bottom: H3K4me3_23d, H3K4me3_52d, K4_diff, H3K27me3_23d, H3K27me3_52d, and K27_diff. Gray bars under sequence read tracks show regions with significant signal (p<1e-6) above the appropriate input background (see Genome Browser and Figure 2).

Validity of data was evaluated by observing gene tracks with expected histone methylation patterns. *ACTIN2* (*At3g18780*, Locus:2093954 ) is highly expressed at both 23 d and 52 d, and strong H3K4me3 marks and no H3K27me3 marks were observed in leaf samples of both ages at this locus (Figure 2A). The *FLC* floral repressor (*At5g10140*) is not expressed in the *fri* (Locus:2127013) mutant Col-0 ecotype [34], [35], and is known to be silenced, in part, by H3K27me3 marks [12]. In both mature and senescent leaf tissue, the *FLC* gene was heavily marked by H3K27me3 (Figure 2B). The H3K4me3 mark was absent except for a small peak that spans the first exon and the beginning of the first intron, and a second small peak that covers the last exon, and may be related to expression of the COOLAIR long non-coding RNA [36].

**Figure 3:**
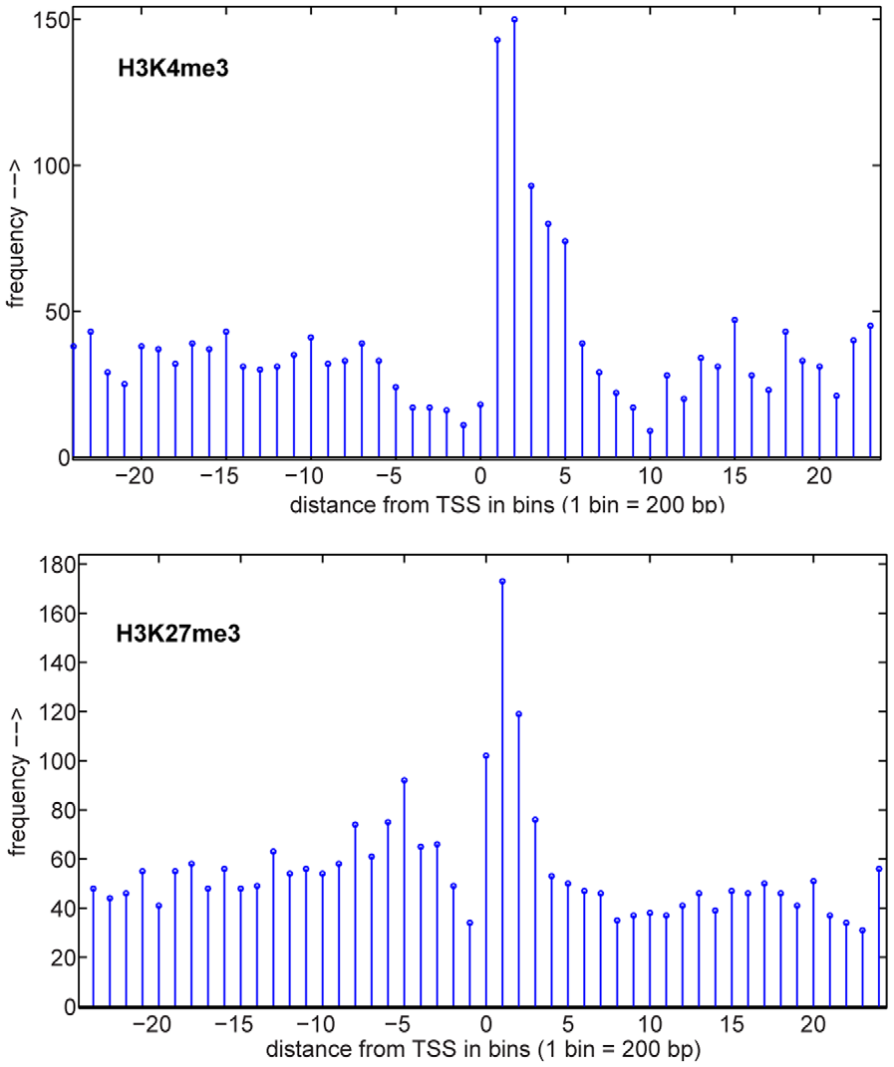
Genic distributions of differential histone marks. Regions with significantly different levels of histone modification were identified by ChIPnorm and placed into 200 bp bins relative to the transcription start site (TSS). A) Differential regions for the H3K4me3 mark are most abundant in the five bins downstream of the TSS. B) Differential regions for the H3K27me3 mark are most abundant at the TSS and the first three bins downstream of the TSS.

K4_diff and K27_diff tracks show regions with significant differences in levels of histone modification as determined by ChIPnorm [37]. Red bars indicate higher levels of histone methylation in the 23 d sample while green bars indicate higher levels of methylation in the 52 d sample in the Genome Browser. The ChIPnorm method was utilized to find differential regions between 23 d and 52 d H3K27me3 and H3K4me3 histone modification ChIP-seq data. ChIPnorm is a two-stage statistical approach to find differentially enriched regions of the genome given two ChIP-seq histone modification libraries. ChIPnorm removes the noise and the bias from two ChIP-seq libraries and normalizes the data to enable a direct comparison between the two libraries to identify differential regions. The first stage removes the regions which have stochastic background noise and local genomic bias, and in the second stage the two libraries are normalized using a quantile normalization procedure and then differential regions are identified. This approach outperformed other approaches in identifying differential regions without bias. Differential regions identified for the H3K4me3 mark were most abundant in the first kilobase (first five 200 bp bins) downstream of the TSS and were depleted in the same region upstream of the TSS (Figure 3A). Differential regions for the H3K27me3 mark were most abundant just downstream of the TSS, but did not show a marked depletion upstream of the TSS (Figure 3B). The location of the H3K4me3 differential regions generally matched the location of H3K4me3 modifications noted previously (20) while the location of H3K27me3 differential regions was further upstream than the previously noted peak of H3K27me3 marks (25% to 75% of gene length) (25).

### H3K4me3 and H3K27me3 marks in Senescence-Regulated Genes

**Figure 4:**
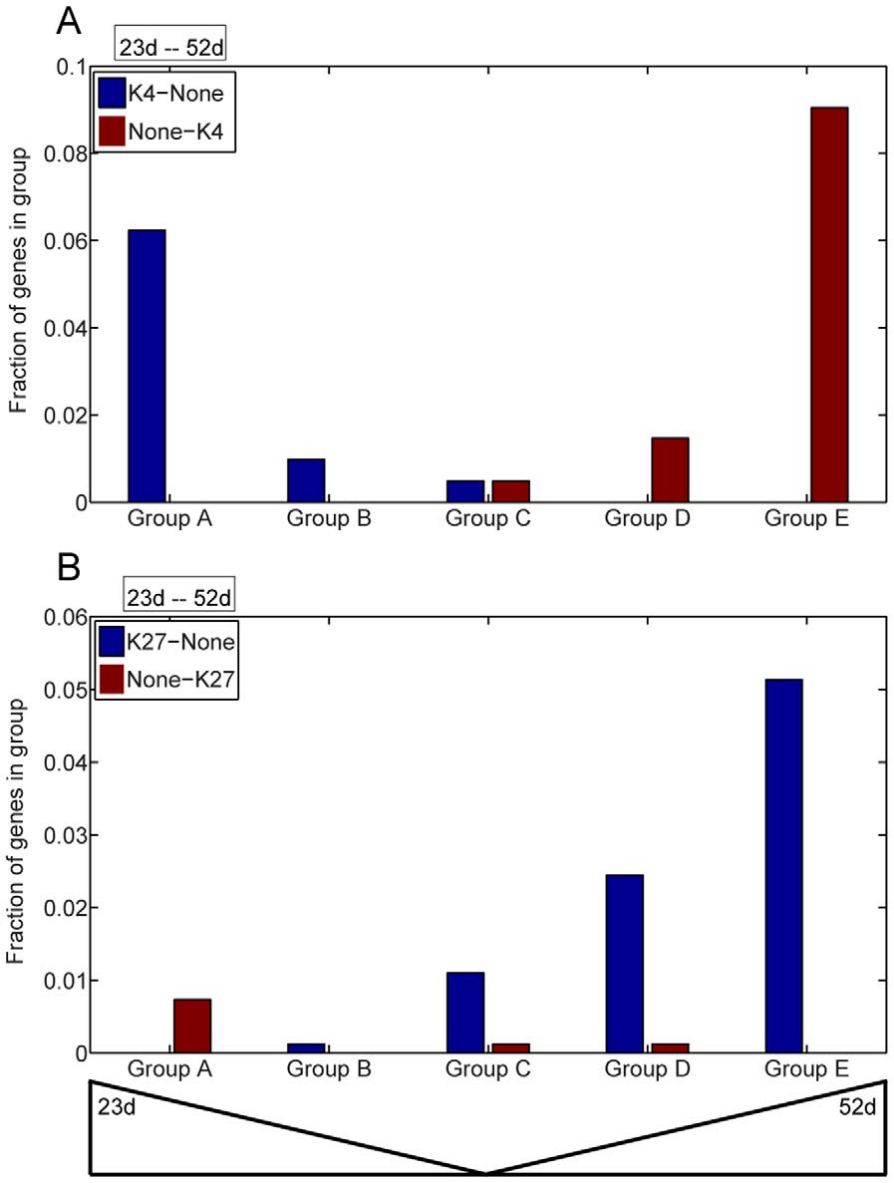
Correlation between regions with differential histone modification and senescence regulated gene expression. 4090 genes were equally partitioned into five groups depending on differential expression in mature (23 d) vs. senescent (52 d) tissue. Group A genes have the highest differential expression at 23 d, group E genes have the highest differential expression at 52 d while group C genes have approximately equal expression at both time points. Genes that were identified as having different levels of histone modification (H3K4me3 in panel A, H3K27me3 in panel B) were then placed into the groups. Genes showing a decrease in the specific histone methylation at 52 d (K4-None or K27-None) are shown in blue while those that showed an increase in the specific histone methylation at 52 d (None-K4 or None-K27) are shown in brick red.

Senescence up-regulated genes (SURGs) and senescence down-regulated genes (SDRGs) were identified from a published microarray analysis using naturally senescent leaf tissue that had lost approximately 25% of total chlorophyll, and was generally similar to the leaf tissue used in this study [4] with the exception that our plants were grown in continuous light at 24°C while the plants analyzed by microarray were grown under 16 hL∶8 hD long-day conditions at 21°C during the day and 19°C at night. To confirm similarity between the two studies, 8 SURGs and 7 SDRGs were analyzed by real-time qPCR using RNA isolated from the same tissue used for ChIP-seq, and results are summarized in Table 1. All 8 SURGs were found to be up-regulated in our senescent leaves while 6 of 7 SDRGs were found to be down-regulated. These data indicate that our tissue is a fair representation of the published microarray results, and permit a larger scale analysis of gene expression and differential histone modifications (Figure 4). A collection of 4090 genes, whose expression values were taken from a supplemental table published in [4], were chosen for this expression analysis. These genes were divided into five groups with group A having the largest differential expression at 23 d (SDRGs), group C having approximately equal expression at 23 d and 52 d, and group E having the largest differential expression at 52 d (SURGs). Genes were categorized as having K4 at both developmental stages (K4-K4), at neither developmental stage (None-None) or only at 23 d (K4-None) or only at 52 d (None-K4). When histone methylation categories were graphed according to expression groups it could be seen that group A and B were enriched for K4-None while groups D and E were enriched for None-K4 (Figure 4A) demonstrating that H3K4me3 modifications play an activating role for SDRGs at 23 d and for SURGs at 52 d. The proportion of genes that were differentially marked was small, with most genes showing None-None (approximately 35% of 4090 genes) or K4-K4 (approximately 60% of 4090 genes). This is similar to observations made in mouse cells [37]. A similar analysis was done for the H3K27me3 modification, and group E genes were most highly enriched for K27-None demonstrating a repressive role for K27 at 23 d. Only a small number of genes showed the None-K27 pattern however these were concentrated in group E genes which do show reduced expression at 52 d (Figure 4B). For H3K27me3, the largest proportion of genes were unmodified (None-None, approximately 90% of 4090 genes), which is also similar to the analysis done for mouse [37]. SURGs (group E) that showed an increase in the H3K4me3 mark in older tissue were subject to GO analysis, and over-represented categories include response to stress and response to water deprivation. SURGs (group E) that showed a decrease in the H3K27me3 mark were over-represented in response to abiotic stress as well as response to water deprivation GO categories. Leaves undergoing senescence do experience oxidative stress and water loss. A small number of genes were shared among the response to water deprivation classification indicating that H3K4me3 increase was coupled to a decrease in H3K27me3 for a subset of genes. SDRGs (group A) that showed a decrease in the H3K4me3 mark were enriched for the response to auxin stimulus category, including four SAUR-like genes (*At5g18030*, *At4g34760*, *At1g29460*, *At5g18060*) and the *AUX1* auxin influx transporter (*At2g38120*). These genes are recognized to be involved in rapid auxin response and cell elongation, and down-regulation during senescence is expected and appears to involve a decrease in the H3K4me3 mark.

**Table 1:** Real-time qPCR measurement of senescence-up-regulated genes (SURGs) and senescence-down-regulated genes (SDRGs) mRNA levels in tissue used for ChIP-seq.

	23 d	52 d
**SURGs**		
At5g45890, SAG12	1	90148
At1g29640	1	635
At3g44300	1	309
At5g13080	1	304
At1g73220	1	113
At2g29460	1	99
At5g42800	1	74
At1g13340	1	16
**SDRGs**		
At2g10940	14116	1
At3g16670	2788	1
At5g26000	70	1
At3g05730	55	1
At5g25460	53	1
At3g27690, Lhcb2.3	25	1
At3g44990	0.4	1

Total RNA was harvested from the same leaves used in the ChIP-seq analysis, and real-time qPCR was performed using *ACT2* as a reference. SURGs and SDRGs were identified from published microarray data [4]. Fold-induction at 52 d for SURGs and at 23 d for SDRGs is reported. Eight of 8 tested SURGs were up-regulated by at least 15-fold, while 6 of 7 tested SDRGs were down-regulated by at least 24-fold.

**Figure 5:**
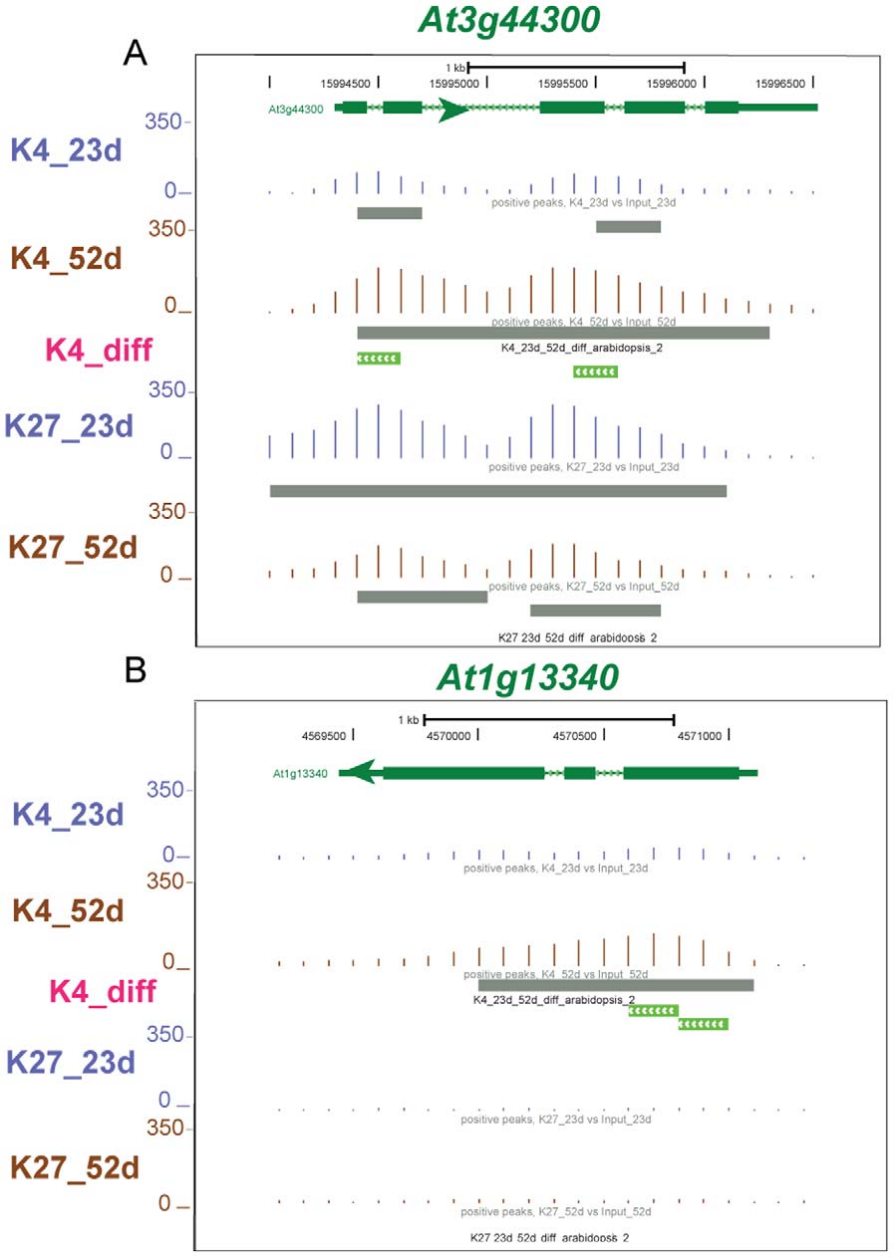
Genome Browser view of two genes that are up-regulated in senescent leaves and show differential histone modification. These GBrowse views are similar to Figure 2, but a new track, K4_diff (pink font) is now shown. The green bars indicate significantly higher histone methylations at 52 d. A) *At4g44300* shows a 300-fold increase in mRNA at 52 d which is accompanied by increased H3K4me3 marks. B) *At1g13340* shows a 16-fold increase in mRNA which is also accompanied by increased H3K4me3 marks.

**Figure 6:**
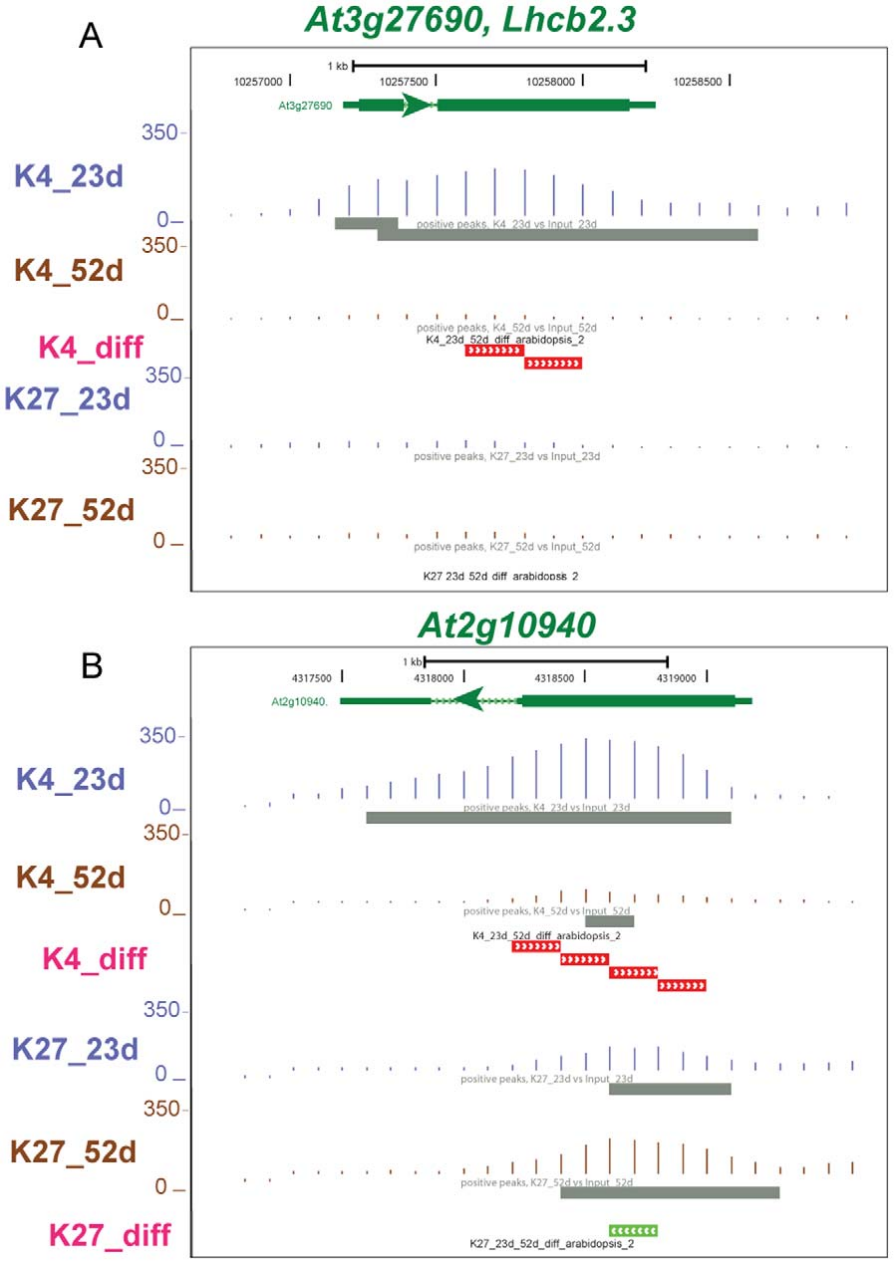
Genome Browser view of two genes that are down-regulated in senescent leaves and show differential histone modification. These GBrowse views are similar to Figure 5, but a new track, K7_diff (pink font), is now shown. The red bars in the K4_diff track indicate significantly higher histone methylations at 23 d while the green bars in K7_diff indicate significantly higher histone methylations at 52 d. A) *At3g27690* or Lhcb2.3, shows a 25-fold decrease at 52 d which is accompanied by a decrease in H3K4me3 marks. B) *At2g10940* shows a 14,000-fold decrease at 52 d which is accompanied by a decrease in H3K4me3 marks and an increase in H3K27me3 marks.

ChIP-seq reads are shown for two SURGs in Figure 5 and two SDRGs in Figure 6. *At3g44300* is up-regulated 309-fold and *At1g13340* is up-regulated 16-fold in older leaves (Table 1). The K4_diff track is visible and colored green for both genes since there is an increased level of the H3K4me3 mark in the 52 d sample. *Lhcb2.3* is down-regulated 25-fold and *At2g10940* is down-regulated 14,000-fold in older leaves (Table 1), and both have K4_diff tracks colored red since there is increased level of the H3K4me3 mark in the 23 d sample. Interestingly, the strongly down-regulated *At2g10940* also shows a significant increase in the H3K27me3 mark in the 52 d tissue shown by the green bars in the K27_diff track.

**Figure 7:**
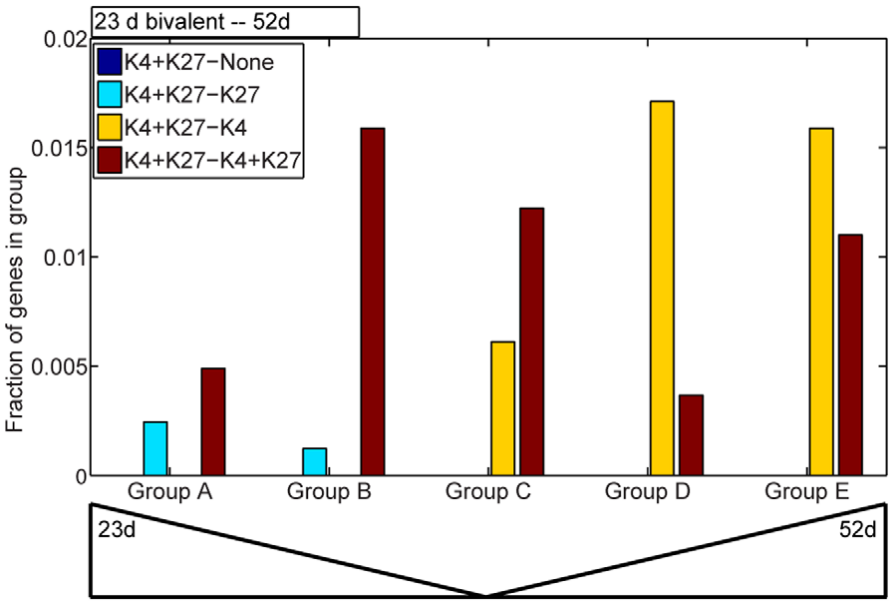
Analysis of bivalently modified genes. Bivalent (H3K4me3 and H3K27me3) modifications were observed for a subset of genes at 23 d. These genes were classified into those that lost both modifications at 52 d (K4+K27- None, dark blue), those that retained the H3K27me3 mark at 52 d (K4+K27 – K27, light blue), those that retained the H3K4me3 mark at 23 d (K4+K27- K4, yellow) and those that retained the bivalent marks (K4+K27- K4+K27, brick red). Genes thus classified were then placed into the five groups shown in Figure 4. Genes that retained the H3K27me3 mark were restricted to groups A and B while genes that retained the H3K4me3 mark were restricted to groups C–E.

A small number of genes showed bivalent H3K4me3 and H3K27me3 modifications at 23 d which became monovalent H3K4me3 or H3K27me3 modifications at 52 d. Genes retaining the activating H3K4me3 marks were in groups C, D and E, while genes retaining the H3K27me3 marks were only in groups A and B (Figure 7). These results provide additional support for an activating role for H3K4me3 and a silencing role for H3K27me3 as leaves age.

**Figure 8:**
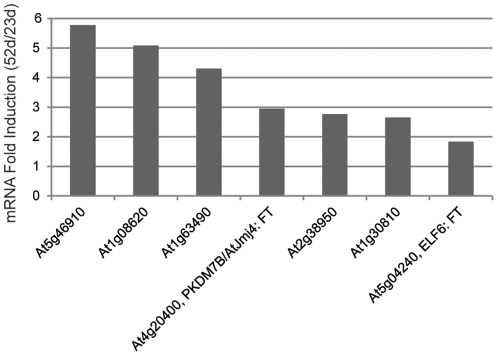
Expression of Arabidopsis *KDM5B*–like genes in senescent leaf tissue. *KDM5B* genes encode H3K4me3 demethylases, and eight KDM5B-like genes were identified in Arabidopsis. The same tissue used in the ChIP-seq analysis was analyzed for expression of the *KDM5B-like* genes using real-time qPCR. *ACT2* was the reference gene and relative expression at 52 d compared to 23 d is shown. Two *KDM5B-like* genes affect flowering time [41], [42], [43], and their published names as well as known target gene are shown. *At2g34880* mRNA was undetectable in both RNA samples, and is not shown.

H3K4me3 demethylation is catalyzed by KDM5B/JARID1B family members [38], and there are 8 potential *KDM5B-like* genes in Arabidopsis [39], [40]. Expression of these eight genes was measured in 52 d and 23 d leaf tissue using real-time qPCR with *ACT2* as the reference. One of the genes, *At2g34880*, had undetectable mRNA levels in both tissues. Six of the remaining 7 genes showed some degree of up-regulation in the senescent leaf tissue with *At5g46910*, *At1g08620*, *At1g63490*, *At4g20400*, *At2g38950* and, *At1g30810* all displaying a greater than 2-fold induction (Figure 8). One of these genes, *At4g20400* has been shown to affect the timing of flowering [41], [42], [43], but the five other *KDM5B-like* genes do not yet have ascribed functions (www.arabidopsis.org).

One other study quantified H3K4me3 marks in senescent Arabidopsis leaf tissue at the *WRKY53* locus (At4g23810, Locus:2128514), and found a small increase in this modification in senescent leaves [18]. Our genome-wide quantitative analysis also showed enriched H3K4me3 marks on the *WRKY53* gene, however no quantitative difference in these marks was observed between the 23 d and 52 d samples (Figure S2). No significant enrichment of H3K27me3 marks was observed for the *WRKY53* gene. In our tissue, *WRKY53* mRNA was only up-regulated by 1.7-fold at 52 d compared to 23 d. The largest increase in *WRKY53* expression likely occurred prior to tissue harvest [44], and this may explain the discrepancy with the previous study.

**Figure 9:**
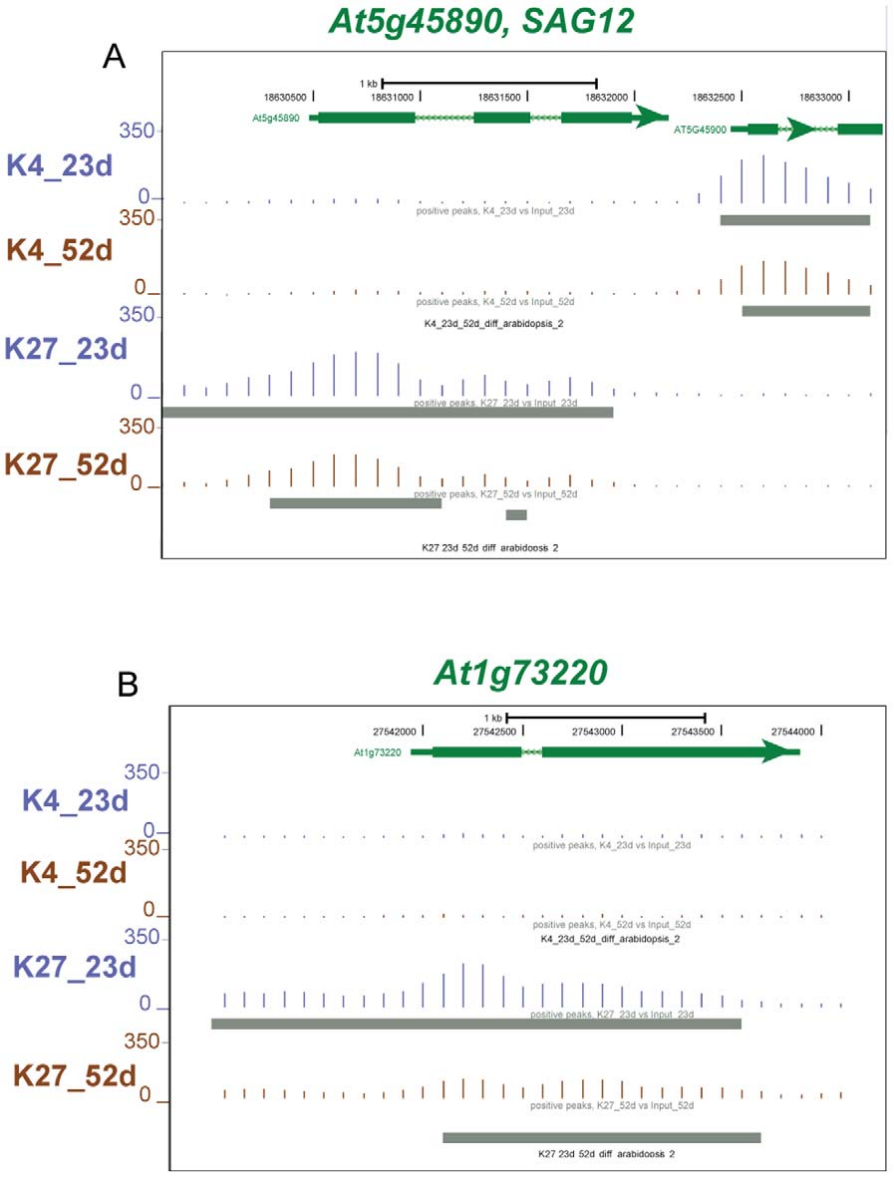
Two genes that are expressed at high levels in senescent tissue lack activating H3K4me3 marks. A) *SAG12* (*At5g45890*) is up-regulated 90,000-fold at 52 d, but is devoid of H3K4me 3 marks. B) *At1g73220* is up-regulated 113-fold at 52 d and likewise shows no H3K4me3 marks. For both genes, H3K27me3 marks are present, but do not show a significant difference between 23 d and 52 d.

Surprisingly, two genes that are strongly up-regulated during senescence did not show expected H3K4me3 histone modification patterns. *SAG12* (*At5g45890*), which encodes a cysteine protease and is considered a molecular marker for senescence, was up-regulated 90,000-fold in the 52 d tissue (Table 1), but this gene was devoid of H3K4me3 marks at both 23 d and 52 d. H3-general sequence reads showed that histones were present in the *SAG12* promoter region (data not shown). In addition, H3K27me3 modifications were found in this region, but trimethylation levels were not significantly different between the two samples as shown by a lack of green or red bars in the K27_diff tracks. The adjacent gene, *At5g45900*, did contain H3K4me3 marks near its TSS indicating there is no general loss of this modification in this region of the genome, rather *SAG12* specifically lacks H3K4me3 marks (Figure 9A). It is unlikely that increased mRNA stability is the cause of increased mRNA levels because the *SAG12* promoter has been shown to activate reporter genes during senescence [45]. A second SURG, *At1g73220*, encodes a carbohydrate transporter, and mRNA levels were increased 113-fold at 52 d (Table 1). This gene also completely lacks the H3K4me3 mark despite high mRNA levels at 52 d (Figure 9B). *At1g73220* does have similar levels of H3K27me3 marks in both mature and senescent leaves indicating that histones are present in this region. These two important exceptions show that the H3K4me3 mark is not required for high levels of gene expression in senescent leaf tissue.

**Figure 10:**
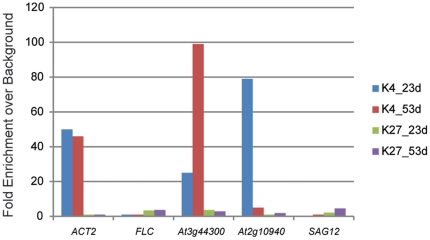
Biological replicate to confirm ChIP-seq data. Plants were grown under similar growth chamber conditions for 23 d and 53 d and subject to ChIP followed by real-time qPCR with primers that were designed to amplify the peaks of histone modification observed with ChIP-seq. The *ACT2* gene showed high enrichment for the H3K4me3 mark in both samples while the *FLC* gene showed some enrichment for the H3K27me3 mark in both samples. Differential H3K4me3 modifications were observed for the SURG *At1g44300* and the SDRG *At2g10940*. In addition, the gain in H3K27me3 marks at 52 d was observed for *At2g10940*. The *SAG12* gene lacked H3K4me3 marks.

A biological replicate was performed utilizing plants grown under similar conditions and harvested at 23 d and 53 d. Nuclei were isolated and ChIP was performed using antibodies from the same company, but different lot numbers. Immunoprecipitated DNA was analyzed by real-time qPCR, and results were similar to ChIP-seq findings (Figure 10). H3K4me3 modifications were associated with *ACT2*, but absent in *FLC*. *At1g44300* (SURG, Figure 5A) showed an increase in the H3K4me3 mark in older leaves while *At2g10940* (SDRG, Figure 6B) showed a decrease in the H3K4me3 mark in older leaves. The H3K4me3 modification was absent in *SAG12* (SURG, Figure 10). The H3K27me3 antibody displayed reduced avidity, and only small amounts of the *FLC* genomic regions were immunoprecipitated. Increased H3K27me3 marks were observed at the *At2g10940* gene at 52 d (Figure 6B), and this similar pattern was observed for the biological replicate (Figure 10).

## Discussion

We report genome-wide patterns of histone H3 trimethylation at lysine 4 and lysine 27 in mature (23 d) and senescent (52 d) Arabidopsis leaves. Senescence is the orderly, final process of leaf development, and this study aimed to determine if there were any epigenetic changes that marked the chromatin of the senescent leaf and if these correlated to changes in gene expression. The H3K4me3 mark is predominantly associated with active genes while the H3K27me3 mark is predominantly associated with silenced, tissue-specific genes. Genome-wide analyses of both modifications have been reported for 10–14 day old seedlings grown *in vitro* on artificial medium that contained sucrose [16], [23], [29], while the data presented in this work catalogue these histone methylations in chromatin from leaves harvested from plants grown on soil at two stages of development: mature, fully-expanded leaves from plants with incipient bolts (23 d) and leaves harvested from plants undergoing natural senescence (52 d). Arabidopsis is a monocarpic plant in which whole-plant senescence follows seed production. Senescent leaves were harvested when approximately half of the siliques were brown, and therefore undergoing the process of whole-plant natural senescence. At this time, the oldest leaves of the rosette were yellow or brown, while the younger, but fully-expanded, leaves of the rosette were still green. These green fully-expanded leaves were harvested for the senescent sample and represent older leaves that will soon become yellow and go through cell death. Similar expression patterns for 15 of 16 genes shown to be up- or down-regulated during senescence in previous studies [1], [4], demonstrated that the 23 d leaf tissue was mature, while the 52 d sample was undergoing natural senescence and allowed a larger scale expression analysis from a separate study to be correlated with these ChIP-seq results.

Correlation of changes in histone modifications to gene expression revealed that SURGs displayed increased H3K4me3 in the senescent tissue while SDRGs showed increased H3K4me3 modifications in the younger tissue. These changes in modification status were only observed in 9% of SURGs from group E and 6% of SDRGs from group A, which although a small fraction, is over 60 genes, and likely more since the analysis was performed with only 4090 genes. Only slight changes in the expression of ten ATX and ATXR genes which encode H3K4 methyltransferases [28], were observed in older leaves [1] indicating that H3K4 trimethylation activity is not transcriptionally regulated. Group A SDRGs that showed a decreased H3K4me3 methylation were enriched in the response to auxin category. Enrichment of auxin-response genes was also noted for the H3K27me3 mark in another study [32] indicating that histone modification may play an important role in the regulation of auxin responses.

For the 4090 genes analyzed by ChIPnorm, approximately 35% of the genes were classified as None-None with respect to H3K4me3 modifications at 23 d and 52 d, and this value was closer to 50% for group E SURGs. This large proportion of SURGs was expressed without significant levels of the activating H3K4me3 mark. Two striking examples are *SAG12* and *At1g73220*, which were both highly expressed during senescence, yet completely lacked H3K4me3 marks. It is possible that other epigenetic marks, such as histone acetylation or cytosine methylation, may be changing as a leaf enters senescence, and these modifications will be evaluated at these two loci in future work. In addition, modifications at enhancers may affect gene expression, and this, too, can be evaluated [46]. H3K4me3-independent gene expression has not been widely reported, however one study demonstrated that the human Autoimmune Regulator (AIRE, NM_000383) protein can activate expression of target genes that lack H3K4me3 marks [47], however induced expression levels were low, which differs from the highly expressed *SAG12* mRNA. Thus, the observation of H3K4me3-independent high expression appears to be novel. The increased expression of *SAG12* and *At1g73220* could be mediated by the respective promoters in a histone-modification-independent manner. The presence of H3K27me3 marks was delayed compared to changes in gene expression for a dexamethasone-responsive *FLC* transgene [34]. In this study, addition of DEX increased *DEX:: FLC* gene expression within 6 h, yet a decrease in H3K27me3 was not observed for 12–24 h. These results show that changes in gene expression can precede changes in histone methylation, however H3K4me3 modifications were not measured in this study.

Six percent of SDRGs displayed a loss of the H3K4me3 mark. H3K4me3 demethylation is catalyzed by KDM5B JmJC-domain containing proteins [38], and 8 putative *KDM5B-like* genes have been identified in Arabidopsis. One of these genes, *At4g20400*, displays H3K4me3 demethylase activity when recombinant protein is purified from *E. coli* [41], [43]. Other *KDM5B-like* genes play roles in flowering [42], brassinosteroid signaling [48] and gametophyte development [49]. Seven of the 8 *KDM5B-like* genes were found to be up-regulated in the 52 d leaf tissue, and six of the genes were up-regulated over 2-fold. Expression of SDRGs is currently being evaluated in T-DNA insertion lines that disrupt senescence up-regulated *KDM5B-like* genes that do not affect the timing of flowering, and higher order mutants will be constructed based on results from single mutant studies.

Differences in trimethylation of H3K27 were also evaluated by ChIPnorm and correlated to gene expression. A decrease in H3K27me3 marks at 52 d was observed for SURGs, but an increase in H3K27me3 at 52 d was only rarely observed for SDRGs. H3K27me3 marks can be removed by the REF6 demethylase [50], which is expressed at both 23 and 52 d (data not shown). The H3K27me3 methylation status was None-None for approximately 90% of the genes in groups A through E indicating this histone methylation is one of many mechanisms that are utilized for the transition into leaf senescence.

This study evaluated H3K4me3 and H3K27me3 histone marks in mature and senescent Arabidopsis leaf tissue using ChIP-seq and ChIPnorm and genome-wide results are displayed on a publicly available Genome Browser web site. Differentially modified regions were identified, and an activating role for H3K4me3 as well as a repressive role for H3K27me3, were revealed. Activating H3K4me3 marks were found to decrease in genes down-regulated during senescence and numerous *KDM5B*-like genes, encoding H3K4me3 demethylases, were found to have increased expression in senescent leaves. Surprisingly, two genes with high levels of expression in senescent leaf tissue lacked activating H3K4me3 marks. Although other histone modifications and DNA methylation need to be tested, these results show that high expression levels can be uncoupled from the H3K4me3 mark in senescent leaf tissue.

## Materials and Methods

### Plant Growth Conditions

*Arabidopsis thaliana* ecotype Col-0 were grown on Sunshine Mix #1 soil under constant illumination (55 µmoles photons m^−2^ sec^−1^) at 24°C in a Percival E36HO light chamber, and fertilized weekly, for the number of days indicated.

### Preparation of Nuclei

Nuclei were isolated from leaf tissue using a modified version of a published protocol [51]. Well-watered plants were placed in the dark overnight to deplete starch reserves. Four g of leaf tissue was loosely packed into a 50 ml conical tube and 37 ml of crosslinking buffer (0.4 M sucrose, 10 mM Tris, pH 8.0, 1 mM EDTA, pH 8.0, 1% formaldehyde, 1 mM PMSF) was added to the tube which was covered with parafilm, pricked with a needle and placed in a vacuum chamber evacuated with the house vacuum line for 10 min. 2.5 ml of 2 M glycine was then added to the tube, and vacuum infiltration continued for 5 min. Leaves were rinsed 3× in cold, deionized water, and blotted dry on Kimwipes. Dried tissue was ground in liquid nitrogen for 2.5 min, and transferred to a 50 ml tube containing 25 ml of cold nuclei isolation buffer (0.25 M sucrose, 15 mM PIPES, pH 6.8, 5 mM MgCl_2_, 60 mM KCl, 15 mM NaCl, 1 mM CaCl_2_, 0.9% Triton X-100, 2 µg/ml pepstatin A, 2 µg/ml aprotinin) and vortexed for 10 sec every min for 15 min, returning sample to ice between vortex steps. The slurry was then filtered through four layers of autoclaved cheesecloth and the filtrate was centrifuged at 11,000 g for 20 min at 4°C. The supernatant was immediately poured off and the green/white pellet was gently resuspended in 2 ml cold nuclei lysis buffer (50 mM HEPES, pH 7.5, 150 mM NaCl, 1 mM EDTA, pH 8.0, 1% SDS, 0.1% Na deoxycholate, 1% Triton X-100, 1 mM PMSF, 1 µg/ml pepstatin A, 1 µg/ml aprotinin). Four ml of ChIP dilution buffer was added to the sample (150 mM NaCl, 16.7 mM Tris, pH 7.5, 3.3 mM EDTA, pH 8.0, 1% Triton X-100, 0.1% SDS, 0.5% Na deoxycholate, 1 mM PMSF, 1 µg/ml pepstatin A, 1 µg/ml aprotinin), and 300 µl aliquots were flash frozen in liquid N_2_, and stored at −80°C.

### Chromatin Immunoprecipitation

Fifty µl of Dynabeads™ Protein G (Invitrogen, Inc) were washed 3× with 500 µl ChIP dilution buffer, and resuspended to 95 µl with ChIP dilution buffer plus 5 µl of antibody [H3K4me3 (Millipore 17-678), H3K27me3 (Millipore 07-449) H3-general (Millipore 17-10046)], and rotated at 4°C for 2 h. After a quick spin, Dyanbeads coupled to antibodies were washed once with 500 µl ChIP dilution buffer and then twice with 500 µl ChIP dilution buffer with 5 mg/ml BSA. After washes, Dynabeads coupled to antibodies were resuspended in 500 µl ChIP dilution buffer with 5 mg/ml BSA and rotated at 4°C for 2 h to saturate non-specific binding sites.

Nuclei were thawed on ice and then transferred to 1.5 ml polymethyl pentene tubes (Diagenode, Inc) and sonicated in a Diagenode BioRupter at 4°C for 30 cycles of 30 sec sonication at the highest setting and 30 sec off. Sonicated nuclei were centrifuged at 14,000 rpm at 4°C for 10 min, and supernatant was transferred to a fresh tube containing 700 µl ChIP dilution buffer on ice. The 1 ml of sonicated nuclei were added to 50 µl of Dynabeads™ protein G that had been washed 3× with 500 µl ChIP dilution buffer, and rotated for 2 h at 4°C in order to pre-clear the nuclei for non-specific binding activity.

Dynabeads coupled to antibody were pulled back, the supernatant was removed, and tubes with beads were placed on ice. Dynabeads from pre-cleared nuclei were pulled back, the pre-cleared nuclei were transferred to the tubes with Dyanbeads coupled to antibody on ice (or an empty tube for the input sample), and tubes were rotated overnight at 4°C.

Beads were washed 3× (500 µl) with three sequential ChIP Wash Buffers (A, B, C). Each wash step was done for 4 min with rotation at RT. ChIP Wash Buffer A: 20 mM Tris, pH 8.1, 150 mM NaCl, 2 mM EDTA, 0.1% SDS, 1% Triton X-100; ChIP Wash Buffer B: 20 mM Tris, pH 8.1, 500 mM NaCl, 2 mM EDTA, 0.1% SDS, 1%Triton X-100; and ChIP Wash Buffer C: 10 mM Tris, pH 8.0, 250 mM LiCl, 1 mM EDTA, 1% Na-deoxycholate, 1% NP-40. Dynabeads were then washed twice in 1 ml 10 mM Tris, pH 7.5, 1 mM EDTA. Dynabeads were then eluted twice each with 100 µl of 100 mM Tris, pH 8.0, 18.75 mM EDTA, 1% SDS at 65°C for 15 min with vortexing every 2 min. Dynabeads were pelleted in a microcentrifuge and the eluants were combined. 200 µL of input sample was removed after overnight incubation without washes. 200 µl of 0.4 M NaCl diluted in TE was added along with 2 µl RNAse A (10 mg/ml), followed by 10 min at 37°C. Crosslinks were then reversed at 65°C for 5 h. Three µl of proteinase K (20 mg/ml) was added and incubated at 55°C for 1 h. DNA was then precipitated overnight using 8 µg glycogen carrier and 850 µl 100% ethanol, washed with 80% ethanol, and the dry pellet was resuspended in 30 µl H_2_O. DNA (2 µl) was quantified using the Quant-IT High Sensitivity Assay (Invitrogen, Inc).

### Library Construction

End repair was performed on 5 ng of ChIP DNA using the End-IT kit from Epicentre Technologies with reactions running for 1 h at RT. DNA was then purified on a Mini-elute spin column (Qiagen) and eluted with 32 µl EB. Adenine overhang reactions were set up with 15 units of Klenow fragment (New England Biolabs) and 0.2 mM dATP and run for 30 min at 37°C. DNA was then purified on a mini-elute spin column and eluted with 18.8 µl of EB. Adaptors were obtained from the USC Epigenome center, and were diluted (1∶40) and added to 2× Rapid Buffer (Enzymatics) and this mixture was added to purified dA-tailed DNA. 3,000 units of Enzymatics T4 DNA Ligase was then added, and ligation was done for 15 min at RT, and promptly stopped by addition of 250 µl PB buffer (Qiagen) and purification on a mini-elute spin column. DNA was eluted twice, once with 20 µl of EB and then with 19.2 µl EB, and eluants were combined. PCR reactions were set up with 2 units of Platinum *Pfx* DNA Polymerase (Invitrogen) with 0.4 mM dNTPs, 2 mM MgSO_4_ and 0.5 mM primers. Amplification conditions were 94°C for 2 min, 98°C for 30 sec followed by 18 cycles of 98°C for 10 sec, 65°C for 30 sec and 72°C for 30 sec followed by 72°C for 4 min. After PCR was complete, the sample was run on a 1.5% agarose TAE gel using loading buffer with no dye. Multiple samples were run on separate gels to prevent cross-contamination. DNA that was clearly separated from the adaptor dimer band was cut from the gel and purified using spin columns (Qiagen). Gel pieces were melted in QC buffer at 37°C to prevent loss of AT-rich fragments. DNA was eluted in 40 µl of EB and quantified as before. DNA was submitted to the USC Epigenome Center for clustering and single-end sequencing analysis on an Illumina HiSeq system. Adaptors and primers are standard Illumina sequences and are listed at http://epigenome.usc.edu/docs/making_libraries/DNA_Library_Protocol_100428.pdf.

### Real-time qPCR

Total RNA was isolated with TRIzol™ reagent (Invitrogen, Inc), and 1 µg of total RNA was used for cDNA synthesis using random hexamer primers and M-MLV reverse transcriptase. The qPCR Design Tool available at Eurofins mwg/Operon was used for qPCR primer design, primers were tested for linearity with different amounts of cDNA, and all r-values for linearity were above 0.9 0.03 ng of ChIP DNA was used as a template for real-time qPCR amplification. Background was defined as the level of H3K4me3 at FLC and the level of H3K27me3 at ACT2. ABSOLUTE™ QPCR SYBR Green PCR mix (Thermo Scientific) was used in 12 µL reactions amplified in a Stratagene MX3000P™ Real-Time PCR system with a 62°C annealing temperature. Real-time primers used in this study are listed in Table S1 along with TAIR Accession numbers.

### ChIP-seq Analysis

Reads were mapped to the TAIR7 genome using MAQ 0.7.1, and only reads that align to a unique position were retained for further analysis. Duplicate reads that map to the same location on the genome were counted once to reduce clonal amplification effects. The genome was tiled with 100 base windows, and each read was extended by 150 bases which adds one count to each window containing a portion of the read. The counts were estimated for the immunoprecipitate sample as well as the input sample, and the total counts of the input sample were normalized to equal those of the immunoprecipitate. The input sample was used to estimate the expected counts in a window, and if these were zero, they were set to the average value for all windows. Finally, the Poisson distribution was used to estimate the probability of observing the immunoprecipitate counts within a window given the expected counts in the input sample window. We considered all windows with p values less than 1e-6 to have significant peaks. By comparing the number of peaks in the comparison of two inputs, to those found by comparing an immunoprecipitated sample to input, we estimate that this threshold corresponds to a 2% false discovery rate.

For ChIPnorm, the fold change threshold τ was set at 2.0, and the ChIP-seq data were binned at 200 bp.

## Supporting Information

Figure S1 **Comparison of sequence reads from H3 general and Input at 23 d.** The genome wide comparison is centered on the TSS and extends −10,000 bp to +10,000 bp. The number of significantly different windows is small (out of approximately 20,000 genes queried) and shows no pattern with respect to gene location. A similar analysis with 52 d H3 general and Input produced similar results (data not shown).
(TIF)

Figure S2 **Genome Browser view of** ***WRKY53*** **histone modifications.** The *WRKY53* gene (*At4g23810*) had significant enrichment of H3K4me3 marks in mature and senescent tissue, however there was no change in the amount of H3K4me3 marks between the two stages of leaf development. This gene was not marked with H3K27me3.
(TIF)

Table S1 **Primers used in real time qPCR analysis.** An annealing temperature of 62°C was used for all primer pairs.
(DOCX)
