# Supplementary figures and images for: Genome-Wide Evaluation of Histone Methylation Changes Associated with Leaf Senescence in Arabidopsis
Source: PLoS One. 2012 Mar 12;7(3):e33151. doi: 10.1371/journal.pone.0033151 (PMC3299739; doi:10.1371/journal.pone.0033151)

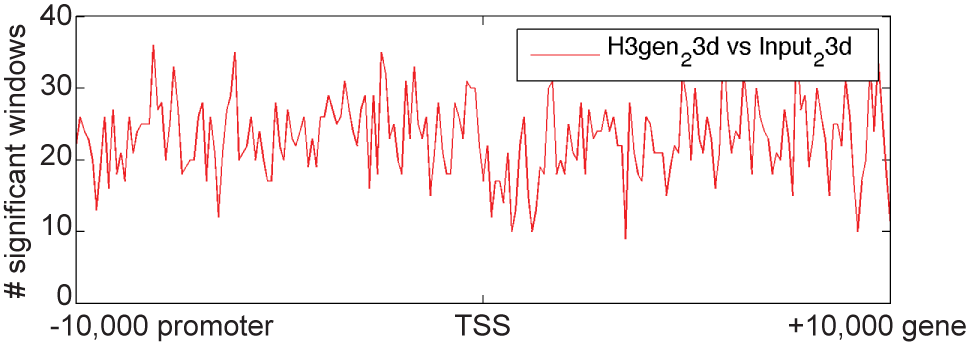

Supplement: Figure S1 — Comparison of sequence reads from H3 general and Input at 23 d. The genome wide comparison is centered on the TSS and extends −10,000 bp to +10,000 bp. The number of significantly different windows is small (out of approximately 20,000 genes queried) and shows no pattern with respect to gene location. A similar analysis with 52 d H3 general and Input produced similar results (data not shown). (TIF) [file pone.0033151.s001.tif]

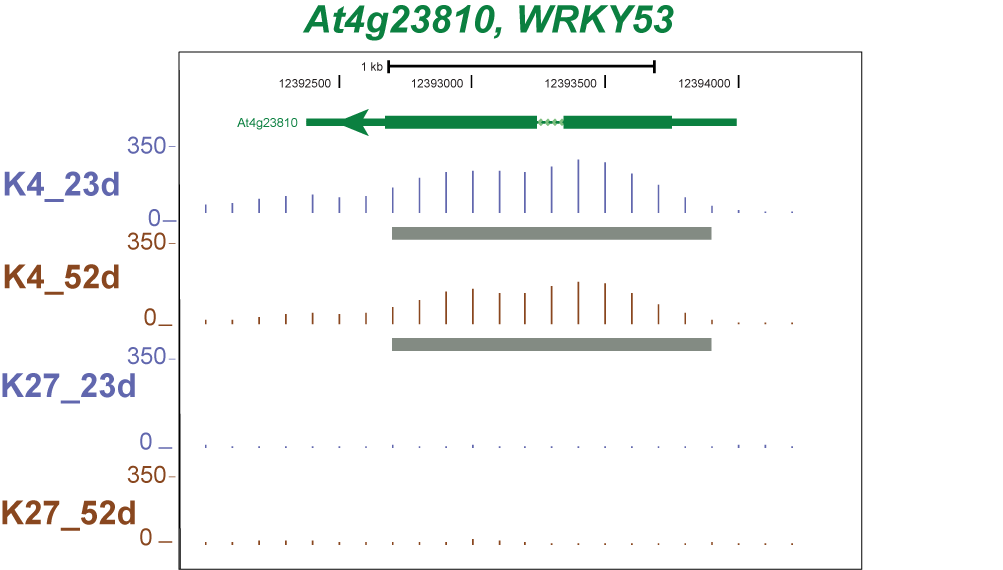

Supplement: Figure S2 — Genome Browser view of WRKY53 histone modifications. The WRKY53 gene (At4g23810) had significant enrichment of H3K4me3 marks in mature and senescent tissue, however there was no change in the amount of H3K4me3 marks between the two stages of leaf development. This gene was not marked with H3K27me3. (TIF) [file pone.0033151.s002.tif]
