# Supplementary material for: Genome-Wide Evaluation of Histone Methylation Changes Associated with Leaf Senescence in Arabidopsis
Source: PLoS One. 2012 Mar 12;7(3):e33151. doi: 10.1371/journal.pone.0033151 (PMC3299739; doi:10.1371/journal.pone.0033151)
Supplement: Table S1 — Primers used in real time qPCR analysis. An annealing temperature of 62°C was used for all primer pairs. (DOCX) [file pone.0033151.s003.docx]

Primers used for SURGs and SDRGs

|  | Fw | Rev |
| --- | --- | --- |
| **SURGs** |  |  |
| At5g45890, SAG12 | CGGCGAATCTACTAACGG | TCAATCCCACACAAACATACACAA |
| At1g29640 | CGAGAAAGAGAATAACCGTCC | CCATCCTCCGTCCAACTATC |
| At3g44300 | TACAACGATACTCCCGCCAC | CACCCCTAAACCAAACCTAAAAC |
| At5g13080 | GTCGTTGTATGCTCCTTTTTTGTC | CCTTTGTTTCTTCCCCTTCTTTTTC |
| At1g73220 | CGAGAAAGGAGAAGCAACAAG | AGCGATAGAGACCAAGAGAG |
| At2g29460 | AGGTTCCGGTTCTTGTCTAC | CTACTCCCTTCTCTGCTTTTG |
| At5g42800 | TTTGTTCGTGCCACCGTTCG | CCTTCCTCAGATAAATCAGCCTTCC |
| At1g13340 | TCAAGAACCAGCGTCAAGCC | GCAAATAGCAAACCCGAAACAG |
| **SDRGs** |  |  |
| At2g10940 | CTTGTCTCTGCACTACCCTC | CCATTCTCTTCTCACTTTTTTCTCC |
| At3g16670 | ACATCCAAACCCAAAGCAAC | ACCACCAAGACCACTCTGAG |
| At5g26000 | GCCCTTTGTTACCCTCTTTC | TCCAGTTCTTTACCCTATCACC |
| At3g05730 | CCATCTGCTTCACCACTCTTC | GCTTCACAGTCTCTTTCCCTAC |
| At5g25460 | ACGACCACTCTCCTTTACCTG | ATGGCACTCTCTTTACCCGC |
| At3g27690 | CCGAAAACACACCATCATACC | TCCCAACATTGCCCATCTAC |
| At3g44990 | ACATCCCTCTATCTCTCAAACAAC | GACATTTCGGTCGCCACTTC |

Primers used for *KDM5B-like* genes

|  | Fw | Rev |
| --- | --- | --- |
| At5g46910 | CTCTTTGCCCGACTCATTC | CGCTACCATCTACATCACAC |
| At1g08620 | GACCCGACTCCACAAACAAC | ACAGATTATGCCTCCTTCAGATG |
| At1g63490 | CGACCAAAGACCAGAATCAG | GCCAACAAAAAGAAGAGAAGAG |
| At1g30810 | CAGACAAGGATAAGGACAAAGAC | TGATGGAGTGAATACGGGAG |
| At4g20400 | ACGCTCCCATCTTTTATCCC | AGCAAGTCAATGAACTGAATCC |
| At2g38950 | GGAGAACAGCCAAAGAAGAG | ACCAATCAAGGGGAGCAAAG |
| At3g48430 | CGCTATTCTCAGTCGCTCTC | AACCTTACCACACCATTTTCC |
| At5g04240 | CGGCAGAGAGAAGAAAGAGAG | GTCCCACATCACCAAACGAG |
| At2g34880 | CCGTTTCATTTTTCTTCGTTACACC | CCTTCTGAGTCTCTGAACAACC |

Primers used in ChIP-real time qPCR

|  | Fw | Rev |
| --- | --- | --- |
| At3g18780-ACT2 | GCGACTTGACAGAGAAGAAC | GAAAGAGCGGAAGAAGATGAG |
| At5g10140-FLC | GGCACCAAAGAAACAAGGC | GAAAATCGACAATCACACAACC |
| At3g44300 | ACCATCCCCGTTTACGACAC | TGCATCAAGAGCTGACCTTTG |
| At2g10940 | TTGAGGGTAGTGCAGAGAC | AGTAAAGATAGGGCTTGGGG |
| At5g45890-SAG12 | AAGGAGGAAAACAATCGCTAC | GCAAACTGATTTACCGCAAG |

**Supplemental Table 1.** **Primers used in real time qPCR analysis.** An annealing temperature of 62^o^C was used for all primer pairs.
